# Virtual Reality Simulation in Undergraduate Health Care Education Programs: Usability Study

**DOI:** 10.2196/56844

**Published:** 2024-11-19

**Authors:** Gry Mørk, Tore Bonsaksen, Ole Sønnik Larsen, Hans Martin Kunnikoff, Silje Stangeland Lie

**Affiliations:** 1 Department of Health Faculty of Health Sciences VID Specialized University Stavanger Norway; 2 Department of Health and Nursing Sciences Faculty of Social and Health Sciences Inland Norway University of Applied Sciences Elverum Norway

**Keywords:** 360° videos, health professions education, virtual reality, usability study, undergraduates, university, students, simulation

## Abstract

**Background:**

Virtual reality (VR) is increasingly being used in higher education for clinical skills training and role-playing among health care students. Using 360° videos in VR headsets, followed by peer debrief and group discussions, may strengthen students’ social and emotional learning.

**Objective:**

This study aimed to explore student-perceived usability of VR simulation in three health care education programs in Norway.

**Methods:**

Students from one university participated in a VR simulation program. Of these, students in social education (n=74), nursing (n=45), and occupational therapy (n=27) completed a questionnaire asking about their perceptions of the usability of the VR simulation and the related learning activities. Differences between groups of students were examined with Pearson chi-square tests and with 1-way ANOVA. Qualitative content analysis was used to analyze data from open-ended questions.

**Results:**

The nursing students were most satisfied with the usability of the VR simulation, while the occupational therapy students were least satisfied. The nursing students had more often prior experience from using VR technology (60%), while occupational therapy students less often had prior experience (37%). Nevertheless, high mean scores indicated that the students experienced the VR simulation and the related learning activities as very useful. The results also showed that by using realistic scenarios in VR simulation, health care students can be prepared for complex clinical situations in a safe environment. Also, group debriefing sessions are a vital part of the learning process that enhance active involvement with peers.

**Conclusions:**

VR simulation has promise and potential as a pedagogical tool in health care education, especially for training soft skills relevant for clinical practice, such as communication, decision-making, time management, and critical thinking.

## Introduction

### Background

Virtual reality (VR)–based training has a generally high acceptance amongst trainees, regardless of the technology limitations, usability challenges, and cybersickness [1]. A systematic review of the effectiveness of VR-based simulation training from the past 30 years showed evidence for VR as useful for training cognitive skills, such as spatial memory, learning and remembering procedures, and psychomotor skills. VR was also found to be a good alternative where regular job training was either impossible or unsafe to implement [1].

Targeted training of health care students is paramount to ensuring the provision of patient-centered care and effective communication with patients and their families. Using 360° videos in VR headsets as a starting point for VR simulation has immense potential for the systematic design of learning experiences and for fostering social and emotional learning through collaborative interactions with students [2-4]. Social and emotional learning concerns the development of emotional intelligence skills, including self-awareness, self-management, social awareness, relationship skills, and responsible decision-making [5]. Further, VR simulation offers an active and engaging approach to learning, which can positively impact the motivation of both teachers and students while enhancing learning opportunities and creativity in the learning process [4,6]. Videos of 360° are prerecorded using an omnidirectional camera that films in every direction. This means that the viewer can look around freely as in other VR experiences, but movement and interaction are limited because the camera only records from one position at a time [4].

To ensure active learning, the vicarious VR learning experiences should be provided with an instructional component of debriefing and peer discussion [7]. According to Biggs et al [8], education is about conceptual change, not just the acquisition of information. Such conceptual change takes place when students work collaboratively and in dialogue with others, both peers and teachers. Good dialogue is an essential element of activities that shape, elaborate, and deepen understanding [8].

VR simulation has the great advantage of facilitating a rich, detailed learning environment through different scenarios that are difficult to replicate in reality (eg, frightening situations) [9]. Considering that most health professions are underpinned by a client-centered philosophy of practice, the use of VR will be more relevant when educators create content that is particularly aligned with each specific education program [10]. Consideration of design issues and student experiences of usability are central for developing VR technologies in medical education programs [11]. According to Nielsen [12], usability is defined by five quality components: (1) how easy it is to accomplish the task, (2) efficiency (when learned), (3) memorability, (4) number of user errors, and (5) satisfaction related to the design. Usability has also been extended to comprise user experience that has a more compelling emotional and motivational effect [13]. We were interested in exploring the usability of a VR simulation program designed for health care students, in particular with a view to perceived learning outcomes and possible enhancement of soft skills such as communication skills and clinical decision making.

Even though recent studies indicate that VR is increasingly being used in higher education for clinical skills training and role-playing among health care students [6,14], there is a research gap concerning the use of VR to facilitate the nontechnical skills inherent in the social and emotional competences [5], such as communication, decision-making, and ethical reflection [15-18]. There is a need for more research on VR simulation in higher education programs within health care [4,19,20]. Further research needs to be directed toward the application of immersive 360° videos and how it is experienced by students in higher education [4,19,20] and studies that explore the usability of VR simulation [2,19].

A recent review conducted on implementation of VR argued that future studies should explore viable approaches to incorporate VR into health professions education [21]. During the last 2 years, there have been published a few qualitative [22-24] as well as quantitative studies on the usability of VR in nursing education [4]. Literature searches in Google Scholar using the search words *usability AND *virtual reality AND *occupational therapy/social education, published after 2022, revealed 1 mixed methods study on the usability in physical therapy education [25]. This search did not identify any existing publications on usability of VR simulation in occupational therapy or social education programs.

The recommendations from earlier research as well as the scarcity of usability studies concerning VR in health education programs suggest that VR implementation processes and the usability of VR simulation programs should be further explored and evaluated.

### Study Aim

This study aimed to explore student-perceived usability of VR simulation in 3 health care education programs in Norway. The research question was: What are the students’ perceptions of the usability of the employed VR simulation and related learning activities, and are there differences in perceptions between students in different study programs?

## Methods

### Design and Study Context

This study is part of a larger interdisciplinary project in a Norwegian university, the Solstien 3 project, with the objective to create a VR simulation that portrays situations future health and social workers might encounter in their professional practice. The purpose was to offer students a safe and controlled environment to practice handling challenging and unexpected scenarios without the risk of compromising the well-being of clients, patients, or themselves. The VR simulation, developed and used for soft skills training, was intended to supplement teaching and field placements [26].

The project has developed a “virtual learning center” accessible on a webpage, featuring portrayals of service recipients (patients or clients) through text and images, along with 360° videos illustrating various scenarios [27]. The design of the VR simulation included scenarios and related discussion tasks based on shared learning outcomes for higher education in Norway [28]. The VR simulation in all education programs commenced with a briefing, followed by students watching the scenario using VR headsets, without any possibility to interact with the actors or the environment. The students subsequently participated in facilitated debrief discussions in groups or conducted planned assignments in groups for peer learning. The debrief was concerned with the ethical issues related to the scenario, as well as communication and interpersonal skills. The VR simulation was conducted as part of the regular curriculum for undergraduate students enrolled in the involved education programs; occupational therapy, social education, and nursing. After graduation, those who have undergone these education programs with satisfactory results are qualified to work as health care personnel in Norway, provided authorization from The Norwegian Directorate of Health.

In the early development phase of the Solstien 3 project, both faculty members’ and students’ experience with the prototype 360° video was investigated. The findings showed the importance of VR being contextualized directly in educational programs to create a safe environment for learning [26]. Further, in a pilot study, the students’ experience of the VR simulation was explored. Findings showed that students experienced VR simulation as valuable as a space for authentic, engaging, and reflective practice, and therefore the students felt prepared for professional practice [29]. The use of 360° videos in combination with group discussions activates social and emotional learning and appears to be promising for enhancing professional learning [30]. The process and content of the Solstien 3 project are described in detail and are accessible through the aforementioned webpage [27].

### Participants

Students from 3 undergraduate health care education programs at the same university were invited to participate in this study. These students, from occupational therapy, social education, and nursing, had VR simulation scenarios that were directly associated with their professional practice (developed by the Solstien 3 project). The VR simulation was therefore carried out in different subject-specific courses in different semesters for these 3 education programs. The data were collected during the academic year of 2022-2023, specifically between October 2022 and May 2023. An overview of the VR simulation and the related learning activities in the 3 education programs are displayed in Table 1, and a description of the scenarios and the students’ tasks is displayed in Multimedia Appendix 1. The social education students were in their second semester in their first year of study, while the occupational therapy students were in their fourth semester in their second year of study. The nursing students were in their fifth and sixth semesters in their third year of study.

**Table 1:** Overview of virtual reality (VR) simulation learning activities in 3 different education programs.

	Occupational therapy	Social education		Nursing
Semester and name of subject	Second year, fourth semester *Participation and belonging* (15 ECTS^a^)	First year, second semester *Social education work processes* (10 ECTS)		Third year, fifth and sixth semesters *Home nursing care* (15 ECTS)
Students in class	38 students	95 students		280 students
Introduction	The simulation started with some initial information in plenum. The students were given a short instruction about the task and shown how to use and adjust the VR headsets	The simulation started with some initial information in plenum. The students were given a short instruction about the task and shown how to use and adjust the VR headsets	The simulation started with some initial information in plenum. The students were given a short instruction about the task and shown how to use and adjust the VR headsets	
Group sizes	VR simulation: 12-15 students Discussion groups: 6-7 students	VR simulation: 14-15 students Discussion groups: 6-8 students		VR simulation: 8-9 students Discussion groups: 8-9 students
Organization of the learning activity	The class was divided into 3 VR simulation groups and 6 peer discussion groups. The students spent approximately 3 hours on the whole session.	The class was structured into 3 cohorts for teaching on different days. Each cohort was divided into 2 VR simulation groups and 5 peer discussion groups. The students spent between 2 and 4 hours on the whole session.		The class was structured into 3 cohorts for teaching on different days. The cohort was divided into approximately 11 VR simulation and peer discussion groups. The students spent 1 hour on the whole session (as a part of a full-day health care simulation program).
Peer debrief	After watching the 360° videos, students were organized into peer discussion groups of 6-7 students. Each group had 1 facilitator.	After watching the 360° videos, students were organized into peer discussion groups of 6-8 students. Only 1 facilitator circulated between the groups.		After watching the 360° videos, students stayed in the same peer groups for discussion and debrief. Each group had 1 facilitator.
Learning outcomes	The debrief is concerned with the ethical issues that arise in the situation, as well as communication and interpersonal skills and a discussion on fall prevention.	The students collaborated to discuss and create a written assignment based on their experiences in the situation. The task involved the identification of possible conflicts of values and target behaviors and a discussion on the choice of appropriate mapping tools.		The debrief is concerned with the ethical issues that arise in the situation and the relationship and communication between the nurse and the patient as well as the family.

^a^ECTS: European Credit Transfer and Accumulation System.

### Measurement

To find the most suitable questionnaire for this study, research work with similar objectives was examined. We found 2 relevant studies related to the usability of VR simulation in nursing education [31,32]. Verkuyl et al [31] developed a questionnaire based on earlier usability testing in the same study context. This questionnaire of 18 items had 2 sections: *perceived ease* *of use* and *perceived usefulness.* The questionnaire was relevant to our study as items considered whether the students found the VR simulation easy to use, as well as whether the learning activity would enhance soft skills such as communication skills and clinical decision-making [31]. The questionnaire of Lee et al [32] was a further refinement of the work of Verkuyl et al [31] and consisted of 17 items to be rated and 7 open-ended questions. Based on these 2 studies, a questionnaire was developed by the research group, containing 17 items to be rated and 2 open-ended questions. The questionnaire also included 6 items assessing student characteristics: study program affiliation (social education, occupational therapy, and nursing), gender (male vs female), age (years), current working status in health care or social services (yes vs no), prior experience using VR technology (yes vs no), and attitude toward VR technology in education (sceptical or indifferent vs positive).

In total, 17 statements related to the students’ perceptions of the usability of VR simulation and the related learning activities were used. Of the total items, 9 concerned perceived ease of use and 8 items concerned perceived usefulness. The participants were instructed to rate their level of agreement (1=disagree, 2=disagree somewhat, 3=unsure, 4=agree somewhat, and 5=agree). Toward the end of the questionnaire, the participants were also asked to evaluate the VR simulation activity on a 1-5 scale (1=poorest evaluation and 5=best evaluation).

The questionnaire also had 2 open-ended questions: *What specifically did you learn from the learning activity (360° video, group discussions, etc.)* and *Do you have other comments about the 360° videos* *and the related learning activities? (Improvements? Other ideas?)*

The translation process was first undertaken by one of the research group members before the resulting translations were discussed and adjusted by the team. The students were invited to respond to a digital questionnaire in their native language (Norwegian) shortly after completing the learning activity.

### Data Analysis

Differences between students enrolled in different education programs were examined with Pearson chi-square tests for categorical variables, and with 1-way ANOVA for continuous variables. To adjust for inflating type I error rates in the multiple comparisons, Tukey honestly significant difference (HSD) correction was applied to the analysis of variance. To analyze the data from the open-ended questions, we used qualitative content analysis [33]. Initially, the responses were thoroughly read to gain a broad understanding, followed by a systematic categorization into distinct codes. This process helped in identifying similarities and differences in the responses. Subsequently, these codes were grouped into 2 relevant categories, which facilitated the identification of recurring patterns focusing on students' experiences of the usability aspect of the VR simulation.

### Ethical Considerations

This study is registered with the Norwegian Centre for Research Data (protocol code 423788). Research ethics were strictly adhered to throughout the study. The learning activity was conducted as a mandatory activity as part of the curriculum for all students in the relevant study programs (n=413). After having conducted the learning activity, the students were invited to complete the questionnaire, which was provided by a QR code in the classroom or a link sent by email. On the first page of the questionnaire, the students received information about the study's purpose and procedures, and that participation was voluntary. The students were also informed that they consented to participate in the study by pressing “go to questionnaire”. In this way, informed consent was obtained from all participants before their involvement. The questionnaire was anonymous, and data cannot be traced back to individual informants. Moreover, the questionnaire did not ask for any sensitive information.

## Results

### Student Characteristics

Overall, 146 students responded to the questionnaire out of a total of 413 invited students (35.4% response rate), representing students of social education (n=74, response rate 77.9%), occupational therapy (n=27, response rate 71.1%), and nursing (n=45, response rate 16.1%). The social education students comprised 50.7% of the sample, while the nursing students comprised 30.8% and the occupational therapy students 18.5% of the sample. Most of the students were women (n=117, 80.1%), while 29 (19.9%) were men, with a larger proportion of younger students in the social education program and a larger proportion of older students in the nursing education program. The sample is described in Table 2.

At the time of the data collection, 105 (71.9%) students worked in health care or social services, and half of the students (n=73) had prior experience using VR technology. Statistically significant differences between the groups were found in the “attitude toward VR in education” and the “evaluation of the VR simulation learning activity.” A larger proportion of nursing students were positive toward VR technology in their education program (n=42, 93.3%), compared with occupational therapy students (n=21, 77.8%) and social education students (n=56, 75.7%). Among the nursing students, 26 (72.2%) gave the highest possible rating (5) when evaluating the VR simulation learning activity. In contrast, this rating was given by 7 (33.3%) of the occupational therapy students and 20 (37%) of the social education students.

**Table 2:** Characteristics of students by study program (N=146)^a^.

Characteristics			All, N		Occupational therapy, n (%)		Social education, n (%)		Nursing, n (%)		*P* value^b^	
**Age (years)**												.009
	18-20	17		2 (7.4)		15 (20.3)		0 (0)				
	21-25	78		16 (59.3)		40 (54.1)		22 (48.9)				
	26-30	32		7 (25.9)		11 (14.9)		14 (31.1)				
	31+	19		2 (7.4)		8 (10.8)		9 (20)				
**Gender**												.75
	Male	29		4 (14.8)		16 (21.6)		9 (20)				
	Female	117		23 (85.2)		58 (78.4)		36 (80)				
**Currently working in health care or social services**												.07
	Yes	105		17 (63)		50 (67.6)		38 (84.4)				
	No	41		10 (37)		24 (32.4)		7 (15.6)				
**Prior experience from using VR^c^** **technology**												.16
	yes	73		10 (37)		36 (48.6)		27 (60)				
	No	73		17 (63)		38 (51.4)		18 (40)				
**Attitude toward VR technology in education**												.048
	Positive	119		21 (77.8)		56 (75.7)		42 (93.3)				
	Skeptical or indifferent	27		6 (22.2)		18 (24.3)		3 (6.7)				
**Evaluation of the VR simulation learning activity^d^**												.002
	1-2	5		3 (14.3)		2 (3.7)		0 (0)				
	3-4	53		11 (52.4)		32 (59.3)		10 (27.8)				
	5	53		7 (33.3)		20 (37)		26 (72.2)				

^a^N=146 unless otherwise stated.

^b^*P*-values are based on Pearson chi-square test.

^c^VR: virtual reality.

^d^n=111, and higher ratings indicate more positive attitudes.

### Perceived Usability of the VR Simulation and Related Learning Activities

In the pairwise comparisons between study programs, using the occupational therapy program as the reference group, the nursing students had the highest mean scores, while the occupational therapy students, for the most part, had the lowest mean scores.

### Students’ Perceptions of Ease of Use

In the pairwise comparisons, statistically significant differences were found on all items related to the perceived ease of use. The highest mean scores for all students were on the items “the pace and the narrative in the 360° video were good” (mean 4.71, SD 0.64) and “the presented situation was realistic” (mean 4.68, SD 0.64). The lowest mean score was found on the item “the audio quality of the 360° video was good” (mean 3.86, SD 1.41), which was also the lowest mean score for both the occupational therapy students (mean 2.81, SD 1.57) as well as the social education students (mean 3.72, SD 1.41) throughout the whole questionnaire.

### Students’ Perceptions of Usefulness

In the pairwise comparisons, statistically significant differences were found on all items related to the perceived usefulness. The highest mean scores for all students were on the item “the 360° video and the related learning activities were useful as a part of my education program” (mean 4.46, SD 0.95). The lowest mean score was found on the item “what I experienced in the 360° video improved my professional competence” (mean 3.93, SD 1.19). The difference between the study programs was rather large, with the occupational therapy students scoring this item on average 1.2 points lower than the nursing students and 0.8 points lower than the social education students. Table 3 displays the total mean scores and the pairwise comparisons of mean scores between study programs.

**Table 3:** Usability of VR^a^ simulation and learning activities, N=140-146.

Statements		Total, mean (SD)	Occupational therapy, mean (SD)	Social education, mean (SD)	*P*^b^ value	Nursing, mean (SD)	*P*^b^ value
**Ease of use**							
	The VR^a^ headset and the showtime app were easy to use	4.45 (0.97)	3.67 (1.36)	4.57 (0.83)	<.001	4.73 (0.62)	<.001
	I received sufficient information and instructions before using the equipment	4.65 (0.81)	4.07 (1.24)	4.77 (0.66)	<.001	4.82 (0.50)	<.001
	It was easy to know what to do	4.43 (0.90)	3.89 (1.01)	4.49 (0.89)	.007	4.68 (0.71)	<.001
	I did not have any technical problems	4.01 (1.38)	3.07 (1.47)	4.25 (1.31)	<.001	4.18 (1.21)	.002
	The presented situation was realistic	4.68 (0.64)	3.89 (0.89)	4.86 (0.42)	<.001	4.87 (0.34)	<.001
	The pace and the narrative in the 360° video were good		4.00 (1.00)	4.86 (0.39)	<.001	4.89 (0.32)	<.001
	The visual quality of the 360° video was good	4.50 (0.96)	3.85 (1.57)	4.64 (0.72)	<.001	4.67 (0.67)	.001
	The audio quality of the 360° video was good	3.86 (1.41)	2.81 (1.57)	3.72 (1.41)	.004	4.71 (0.59)	<.001
	I would like to see more scenarios together with related learning activities	4.55 (0.97)	4.19 (1.27)	4.51 (1.00)	.28	4.82 (0.58)	.02
**Usefulness**							
	What I experienced in the 360° video was useful for my future professional practice	4.43 (0.99)	4.00 (1.18)	4.45 (0.97)	.11	4.67 (0.82)	.02
	What I experienced in the 360° video was useful in improving my communication skills	3.95 (1.34)	3.50 (1.53)	3.80 (1.29)	.57	4.45 (1.15)	.01
	What I experienced in the 360° video improved my professional competence	3.93 (1.19)	3.15 (1.43)	3.94 (1.10)	.006	4.39 (0.92)	<.001
	This 360° video was useful as part of my education program	4.38 (1.03)	3.70 (1.38)	4.40 (0.93)	.005	4.75 (0.69)	<.001
	The learning activities were useful for my future professional practice	4.37 (1.01)	3.56 (1.34)	4.42 (0.91)	<.001	4.78 (0.60)	<.001
	The learning activities were useful for improving my communication skills	4.17 (1.12)	3.62 (1.33)	4.08 (1.13)	.14	4.62 (0.75)	<.001
	The learning activities improved my professional competence	4.15 (1.08)	3.13 (1.26)	4.26 (1.00)	<.001	4.53 (0.67)	<.001
	The 360° video and the related learning activities were useful as a part of my education program	4.46 (0.95)	3.85 (1.29)	4.51 (0.88)	.004	4.75 (0.62)	<.001

^a^VR: virtual reality.

^b^*P*-values are based on pairwise comparisons between study programs, with 1-way analyses of variance using the Tukey HSD correction. The occupational therapy program is used as reference group.

### Open-Ended Questions

Out of 146 participants, 110 answered one or both open questions. There were a total of 135 qualitative responses, which were analyzed. Two categories were created based on the responses, focusing on “perceived learning” and “difficulties and recommendations.”

### Perceived Learning

The participants described learning outcomes related to soft skills such as conflict management, reflection, emotion regulation, and non-verbal and verbal skills. Furthermore, they also emphasized professional conduct such as empathy, client-centered practice, and a focus on family involvement. The participants found the following debrief and group discussion particularly useful in their learning experience. One participant expressed it as follows:

With this type of learning, you are better prepared for clinical practice and the learning curve is steep when we reflect together. It was realistic and touched a lot of emotions. Solutions were discussed together. I think this type of learning is extremely beneficial.

The fact that this learning took place in a safe environment was also highlighted as positive by several of the participants. Several of the participants answered the open-ended questions with few words in a general, but positive, manner. For example, the learning activity was cool, exciting, fun, realistic, and relevant for clinical practice. One participant expressed it as follows:

Being a fly on the wall in such a situation was very enlightening.

Some participants also answered that this learning activity was a new type of learning and that the website was particularly useful for getting to know the material more thoroughly.

### Difficulties and Recommendations

The participants' experience of difficulties related to the learning activity mostly concerned either technical or organizational problems. Particularly poor sound quality and the need for noise-reducing headsets were highlighted as technical issues. On the other hand, organizational problems revolved around too many students in the groups and that the preparations could have been more informative, especially for the students with no experience with VR simulation. Two participants reported discomfort related to a rather unpleasant scenario that they found them “in the middle of,” and one of them wrote:

…for me, who is a little light sensitive etc., I had great difficulty having the screen so close to my face. The experience was simply quite unpleasant and triggered a headache.

The participants recommended other types of cases and scenarios involving complex choices and interactive roles in the VR simulation, such as:

…options where you can “decide” the actions further.

It was also desirable that the 360° videos would be more available for self-training and that the students could access more VR simulations at their educational program.

## Discussion

### Overview

This study aimed to explore student-perceived usability of VR simulation, including 360° videos in VR headsets and related learning activities, in 3 health care education programs in Norway. All in all, high mean scores indicate that the students experienced the VR simulation and the related learning activities as very useful. Nevertheless, our findings showed that the nursing students were most satisfied with the usability of the VR simulation, while the occupational therapy students were least satisfied. The qualitative data from the 2 open-ended questions confirmed the findings from the questionnaire, but also illuminated other factors regarding the usability of VR simulation and the related learning activities.

### Differences Between the Study Programs

The nursing students in this study were third-year students with previous experience with other simulations, practical skill training, and clinical practice. They had considerably more experience than the first-year social education students. The second-year occupational therapy students had some prior experience with skill training and clinical practice, but not with simulation. Saab et al [22] emphasized in particular that in order to succeed, the key stakeholders, including students and educators, need to be trained in the use of VR prior to implementing VR simulation. The differences in VR experience among the students prior to this learning session may contribute to explaining why the nursing students scored the usability of the VR simulation significantly higher than students in the other 2 educational programs. They may be due to the novelty effect, where novice VR users tend to focus on managing the hand controllers and exploring the VR environment, rather than focusing on the proposed learning tasks [34].

The key findings in the systematic review of Woon et al [3] illuminate that VR increases nursing students’ engagement in the learning experience when allowing them to participate in activities that are close to reality and when implementing an effective training regime consisting of short interval training (≤30 min each) for a number of sessions. In our study, the nursing students had this learning activity as part of a larger session with a multiple number of short interval trainings. The larger number of trainings for the nursing students and their embedding the VR simulation into a more comprehensive learning activity may contribute to explaining their higher scores on usability and relevance. Also, the nursing program had the smallest groups in the VR simulation (up to 9 students), while both the social education program and the occupational therapy program reported difficulties with instruction and initiation of VR simulation in somewhat larger student groups (up to 15 students). The occupational therapy program experienced technical problems, particularly with sound control, which may also be reflected in the low mean scores from the survey. Educators at the social education program experienced difficulties with one of the cohorts, due to students having little to no prior experience using VR technology. Noble et al [35] emphasized that one should not take students’ knowledge and skills in using VR equipment for granted. However, their study mirrors our results, with approximately half of the students having no experience with VR even though the education program facilitates the use of VR equipment free of charge.

Group differences related to study progression, previous experience with VR simulation, as well as how the learning activity was structured, both according to group size and how many facilitators were available for the students, can partly explain why students scored the usability differently. A valuable recommendation, consistent with previous research [36], is that smaller student cohorts and having a sufficient number of staff available are important so that the students can receive sufficient help at the right time.

### Practicing Clinical Skills With Realistic Scenarios in a Safe Learning Environment

Our findings in both qualitative and quantitative data showed that the students perceived the scenarios to be realistic and useful for clinical practice. Also, several students shared in the open-ended responses that they experienced the VR learning activity as a safe way to practice their clinical skills. The sober and realistic nature of the scenarios depicting real-world situations appears to be beneficial for students’ skill acquisitions with relevance for clinical practice [14,37-39]. In addition, the open-ended responses indicate that students were emotionally touched and had an increasing engagement with this learning activity. This is beneficial, as increased emotions and engagement in VR simulation can enhance the learning processes [9]. In the development of the prototype for the 360° videos, students experienced the VR simulation to be genuine and realistic due to actors using direct eye contact with the camera. Furthermore, the importance of having a safe learning environment was highlighted, especially when experiencing strong emotions [26]. Although the learning environment was described as safe by many students, a few of them pointed out that they found the VR simulation uncomfortable. Cybersickness can influence the learners’ attitude toward the technology negatively and has been correlated with poorer learning outcomes [40]. For these students in particular, it will be crucial to facilitate the VR simulation to avoid discomfort. To reduce the discomfort among individuals experiencing it, one suggestion is to adapt the VR simulation to a standard desktop [36]. Nevertheless, we have reason to believe that VR simulation with realistic scenarios is an appropriate learning activity to precede clinical practice. Furthermore, this type of teaching has a particular value as students can practice skills needed in a difficult situation but still feel safe in a peer learning process.

### Student Active Learning in Peer Debrief and Group Discussions

Our findings based on both qualitative and quantitative data showed that the students perceived the related learning activities useful for their future professional practice. Many students highlighted the group discussions in the debriefing sessions as particularly beneficial, which is consistent with previous research [7,17,26]. These methods are known to enhance learning by allowing students to actively process information, engage in critical thinking, and articulate their understanding or questions [41,42]. Debrief sessions, in particular, offer a reflective space where students can consolidate their learning, address misconceptions, and gain insights from peers and instructors. This discussion contributes to the larger conversation about active learning in education. Active learning, characterized by student participation and engagement, has been increasingly recognized for its effectiveness in promoting deeper understanding and retention of knowledge [43]. However, some of the students remarked that they were passive observers of the 360 video. They suggested including interactivity in the VR simulation for a better learning experience. This aligns with a Canadian study underscoring the benefits of active participation in VR simulation for occupational therapy students [10]. The learning activity in our study still required active involvement in the debrief discussion where students reflected and engaged with the teaching material together with their peers. A suitable alternative could have been active participation in the VR environment, where students would have the opportunity to interact with the virtual environment, make decisions, and influence the outcome of the scenarios. This could lead to a more engaging learning experience, probably more appropriate for training soft skills such as decision-making, time management, and critical thinking.

Our findings have important implications for educational practices. They suggest that incorporating structured group discussions and debriefing sessions can significantly enhance the VR learning experience by allowing students to engage with and process the learning material related to situations they might encounter in a real-world setting. In addition, VR simulations with the student in an active role will probably increase students' motivation and promote learning.

### Study Limitations and Suggestions for Future Research

The study is based on self-report data only. The response rates among both occupational therapy and social education students were high with 71% (n=27) and 78% (n=74). In contrast, the response rate among the nursing students was only 16% (n=45), and, therefore, the findings must be interpreted with caution. Further to this, there may be a response bias, especially among the nursing students, a group possibly consisting of particularly motivated and high-achieving respondents. This may be one important reason why students in this group were more satisfied with the usability of this learning activity. Differences between the student groups may also have been caused by varying levels of VR experience and competence among the faculty facilitating the relevant sessions. While we have the impression that the faculty in the nursing program had more experience with VR compared to their counterparts in the other education programs, we did not collect data from faculty that could support or contradict this view. Thus, we are unable to assess the possible impact of faculty’s VR experience and competence on the students’ experience, and we suggest that future studies include this information.

Other methodological issues concern that the VR simulation and learning activity was carried out at different year levels among the 3 health care education programs and the presence of age differences between students in the different study programs. Higher age and maturity among the participating nursing students may have resulted in higher levels of satisfaction, while these results may not be representative of differences in the study population. We also note that there might be different “group cultures” related to the evaluation of teaching in different groups of students, essentially meaning that similar experiences are systematically given dissimilar ratings across groups. Thus, there is a possibility that the students’ expressed levels of satisfaction differed more than their experienced levels of satisfaction.

### Conclusions

In summary, the students in all 3 educational programs perceived the high usability of the VR simulation and the related learning activities. Nevertheless, the nursing students were most satisfied, while the occupational therapy students were somewhat less satisfied. By using realistic scenarios, health care students can be prepared for complex clinical situations in a safe environment. Group discussions in the debriefing sessions are a valuable part of the learning process and enhance active involvement with peers. Particular attention should be given to the organization of the VR simulation with small student groups and a sufficient number of involved educators so that students receive guidance when problems occur. These findings contribute to the overall growing evidence showing that VR simulation has promise and potential as a pedagogical tool in health care education, perhaps particularly for training soft skills such as communication, decision-making, time management, and critical thinking—skills that are unquestionably relevant for clinical practice. However, future studies need to expand beyond the pilot phase to study if VR simulation exceeds the value of traditional teaching methods.

## Appendix

Multimedia Appendix 1 Descriptions of scenarios and tasks.

## Abbreviations

HSD: honestly significant difference
VR: virtual reality

## Data Availability

The datasets used and analyzed during the current study are available from the corresponding author on reasonable request.

## References

[1] Renganayagalu SK, Mallam SC, Nazir S (2021). Effectiveness of VR head mounted displays in professional training: a systematic review. Tech Know Learn.

[2] Radianti J, Majchrzak TA, Fromm J, Wohlgenannt I (2020). A systematic review of immersive virtual reality applications for higher education: design elements, lessons learned, and research agenda. Comput Educ.

[3] Woon APN, Mok WQ, Chieng YJS, Zhang HM, Ramos P, Mustadi HB, Lau Y (2021). Effectiveness of virtual reality training in improving knowledge among nursing students: a systematic review, meta-analysis and meta-regression. Nurse Educ Today.

[4] Haugan S, Kværnø E, Sandaker J, Hustad JL, Thordarson GO, Akselbo I, Aune I (2023). Playful learning with VR-SIMI model: the use of 360-video as a learning tool for nursing students in a psychiatric simulation setting. How Can We Use Simulation to Improve Competencies in Nursing?.

[5] Conley CS, Durlak JAD, Weissberg CE, Gullotta RP, Thomas P (2015). SEL in higher education. Handbook of Social and Emotional Learning: Research and Practice.

[6] Choi J, Thompson CE, Choi J, Waddill CB, Choi S (2022). Effectiveness of immersive virtual reality in nursing education: systematic review. Nurse Educ.

[7] Luo H, Yang T, Kwon S, Li G, Zuo M, Choi I (2021). Performing versus observing: investigating the effectiveness of group debriefing in a VR-based safety education program. Comput Educ.

[8] Biggs J, Tang C, Kennedy G (2022). Teaching for Quality Learning at University. 5th ed.

[9] Allcoat D, von Mühlenen A (2018). Learning in virtual reality: effects on performance, emotion and engagement. Res Learn Technol.

[10] Kim J, Nowrouzi-Kia B, Ho ES, Thomson H, Duncan A (2023). Appraising occupational therapy students' perceptions of virtual reality as a pedagogical innovation. Comput Educ: X Real.

[11] Atas G, Topalli D, Cagiltay N (2023). A systematic review of virtual reality and user experience in medicine.

[12] Nielsen J (2012). Usability 101: introduction to usability.

[13] Lewis JR (2014). Usability: lessons learned … and yet to be learned. Int J Hum-Comput Interact.

[14] Beverly E, Rigot B, Love C, Love M (2022). Perspectives of 360-degree cinematic virtual reality: interview study among health care professionals. JMIR Med Educ.

[15] Plotzky C, Lindwedel U, Sorber M, Loessl B, König P, Kunze C, Kugler C, Meng M (2021). Virtual reality simulations in nurse education: a systematic mapping review. Nurse Educ Today.

[16] Bracq MS, Michinov E, Jannin P (2019). Virtual reality simulation in nontechnical skills training for healthcare professionals: a systematic review. Simul Healthc.

[17] Coyne E, Calleja P, Forster E, Lin F (2021). A review of virtual-simulation for assessing healthcare students' clinical competency. Nurse Educ Today.

[18] Jiang H, Vimalesvaran S, Wang JK, Lim KB, Mogali SR, Car LT (2022). Virtual reality in medical students' education: scoping review. JMIR Med Educ.

[19] Blair C, Walsh C, Best P (2021). Immersive 360° videos in health and social care education: a scoping review. BMC Med Educ.

[20] Cypress BS, Caboral-Stevens M (2022). "Sense of presence" in immersive virtual reality environment: an evolutionary concept analysis. Dimens Crit Care Nurs.

[21] Lie SS, Helle N, Sletteland NV, Vikman MD, Bonsaksen T (2023). Implementation of virtual reality in health professions education: scoping review. JMIR Med Educ.

[22] Saab MM, McCarthy M, O'Mahony B, Cooke E, Hegarty J, Murphy D, Walshe N, Noonan B (2023). Virtual reality simulation in nursing and midwifery education: a usability study. Comput Inform Nurs.

[23] Andreasen EM, Høigaard R, Berg H, Steinsbekk A, Haraldstad K (2022). Usability evaluation of the preoperative ISBAR (identification, situation, background, assessment, and recommendation) desktop virtual reality application: qualitative observational study. JMIR Hum Factors.

[24] Mäkinen H, Haavisto E, Havola S, Koivisto JM (2023). Graduating nursing students' user experiences of the immersive virtual reality simulation in learning - a qualitative descriptive study. Nurs Open.

[25] Hartstein AJ, Verkuyl M, Zimney K, Yockey J, Berg-Poppe P (2022). Virtual reality instructional design in orthopedic physical therapy education: a mixed-methods usability test. Simul Gaming.

[26] Lie SS, Røykenes K, Sæheim A, Groven KS (2023). Developing a virtual reality educational tool to stimulate emotions for learning: focus group study. JMIR Form Res.

[27] Solstien 3.

[28] National regulations relating to a common curriculum for health and social care education.

[29] Lie SS, Alvestad RW, Helle N, Vikman MD, Dahl-Michelsen T (2024). Exploring VR simulation in healthcare and social work education: students’ experiences with VR simulation as preparation for professional practice. Uniped.

[30] Helle N, Vikman MD, Dahl-Michelsen T, Lie SS (2023). Health care and social work students' experiences with a virtual reality simulation learning activity: qualitative study. JMIR Med Educ.

[31] Verkuyl M, Romaniuk D, Mastrilli P (2018). Virtual gaming simulation of a mental health assessment: a usability study. Nurse Educ Pract.

[32] Lee Y, Kim SK, Eom MR (2020). Usability of mental illness simulation involving scenarios with patients with schizophrenia via immersive virtual reality: a mixed methods study. PLoS One.

[33] Graneheim UH, Lundman B (2004). Qualitative content analysis in nursing research: concepts, procedures and measures to achieve trustworthiness. Nurse Educ Today.

[34] Miguel-Alonso I, Checa D, Guillen-Sanz H, Bustillo A (2024). Evaluation of the novelty effect in immersive virtual reality learning experiences. Virtual Real.

[35] Noble SM, Saville JD, Foster LL (2022). VR as a choice: what drives learners’ technology acceptance?. Int J Educ Technol High Educ.

[36] Saab MM, Hegarty J, Murphy D, Landers M (2021). Incorporating virtual reality in nurse education: a qualitative study of nursing students' perspectives. Nurse Educ Today.

[37] Coyne E, Frommolt V, Rands H, Kain V, Mitchell M (2018). Simulation videos presented in a blended learning platform to improve Australian nursing students' knowledge of family assessment. Nurse Educ Today.

[38] Wang W, Liang Z, Blazeck A, Greene B (2015). Improving Chinese nursing students' communication skills by utilizing video-stimulated recall and role-play case scenarios to introduce them to the SBAR technique. Nurse Educ Today.

[39] Massey D, Byrne J, Higgins N, Weeks B, Shuker MA, Coyne E, Mitchell M, Johnston A (2017). Enhancing OSCE preparedness with video exemplars in undergraduate nursing students. a mixed method study. Nurse Educ Today.

[40] Polcar J, Horejsi P (2015). Knowledge acquisition and cyber sickness: a comparison of VR devices in virtual tours. MM Sci J.

[41] Luctkar-Flude M, Tyerman J, Verkuyl M, Goldsworthy S, Harder N, Wilson-Keates B, Kruizinga J, Gumapac N (2021). Effectiveness of debriefing methods for virtual simulation: a systematic review. Clin Simul Nurs.

[42] Gardner R (2013). Introduction to debriefing. Semin Perinatol.

[43] Børte K, Nesje K, Lillejord S (2020). Barriers to student active learning in higher education. Teach High Educ.

