# Supplementary material for: Virtual Reality Simulation in Undergraduate Health Care Education Programs: Usability Study
Source: JMIR Med Educ. 2024 Nov 19;10:e56844. doi: 10.2196/56844 (PMC11615562; doi:10.2196/56844)
Supplement: Multimedia Appendix 1 [file mededu_v10i1e56844_app1.pdf]

Multimedia Appendix 1

	Get to know the people	Student tasks	Video links	Video still picture
**OCCUPATIONAL THERAPY**	Einar is a man in his fifties. He previously worked as a long-distance lorry driver. Six months ago, Einar had a stroke and became dependent on a wheelchair. He has therefore moved to a care home. Einar has been highly motivated in his training and rehabilitation. One of the goals he has set himself is to return to the stands to watch his local football team play. Recently, however, he has lost some of his motivation to exercise, and has spent a lot of time at home.	You are a clinical placement student, and today you will accompany your placement supervisor to a follow-up of Einar.	Intro/brief: https://vimeo.com/724379051/abb806dac6<br><br>Scenario: https://vimeo.com/722086188/d32ac0e7e6	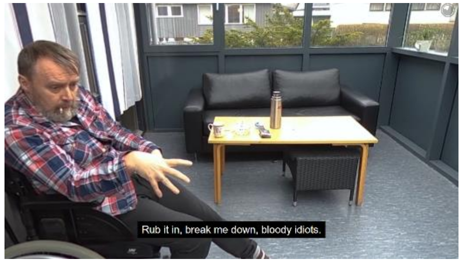
**SOCIAL EDUCATION**	Eva is a young, 20-year-old woman who has recently moved into adapted housing with 24-hour care. Until recently, she lived at home with her parents. She likes to do puzzles and to use her iPad and such like without distractions. Eva has autism and an intellectual disability and needs a fixed framework and predictability in her everyday life. She is wary of new people, does not like loud noises and can react negatively to unfore-seen events. Eva does not have verbal language but can communicate to some degree through signing. She sometimes exhibits challenging behavior. She has self-care abilities in some activities of daily living but may need verbal guidance.	You are a clinical placement student, and today you will accompany your placement supervisor on a visit to Eva. She is sitting in the common room in the residence. Per is also there. He has lived in the residence for several years and is very fond of loud music.	Intro/brief: https://vimeo.com/724378779/d82e35316e<br><br>Scenario: https://vimeo.com/722085866/40486c2e30	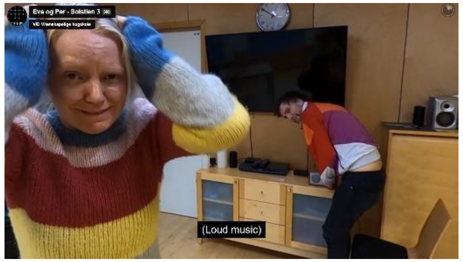
**NURSING**	Gudrun (age 71) is a former teacher. She has 4 adult children: 3 daughters and a son. Gudrun is very fond of nature and the outdoors. In her younger years, she was also an active runner, and among other things, she participated in the Oslo marathon a number of years ago. Gudrun's youngest daughter, Hilde, is worried and tired. She has been taking care of Gudrun for a long time and has watched her get progressively worse. Hilde tries to motivate Gudrun to look after herself, but to little avail. Gudrun lies on the sofa a lot, does not eat well and is drinking more and more alcohol whilst also being prescribed strong medication.	You are a clinical placement student at the home care service. They have received a report of concern from the daughter and is now visiting Gudrun to make an initial assessment.	Info/brief: https://vimeo.com/724378058/680ba38bbc<br><br>Scenario: https://vimeo.com/722085172/98fbbd70df	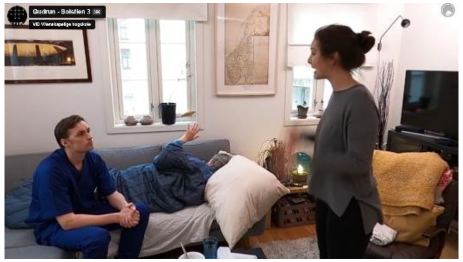
